# Kinetics of thermal degradation of raw lacquer enhanced by formaldehyde urea prepolymer

**DOI:** 10.1038/s41598-023-28787-7

**Published:** 2023-01-30

**Authors:** Qiang Xiao, Yanjun Cao, Wenyu Zheng, Tianle Hou, Shuhang Gao, Jianhua Lyu, Hui Xiao, Yuzhu Chen, Ming Chen

**Affiliations:** 1 College of Forestry, Sichuan Agricultural University, NO. 211, Huiming Road, Wenjiang District, Chengdu City, 611130 Sichuan Province China; 2 Wood Industry and Furniture Engineering Key Laboratory of Sichuan Provinvial Department of Education, Sichuan Agricultural University, Chengdu, 611130 Sichuan China

**Keywords:** Polymers, Composites

## Abstract

In this study, formaldehyde-urea prepolymer (FUP) were synthesized, which were used to modify the raw lacquer (RL) and this composition named LF, while the basic properties of the RL were tested. Thermal gravimetric (TG) analysis and scanning electron microscopy (SEM) were used to analyze the degradative characteristics and the surface morphology of RL before and after modification. The result indicated that FUP can significantly improve the performance of RL. The drying time of the LF is significantly shortened, the gloss, the pencil hardness, and the impact performance are significantly enhanced at the same time. TG analysis and thermal decomposition kinetics analysis illustrated that the thermal stability and the activation energy of LF2 were stronger than that of RL. In addition, SEM analysis illustrated that the surface smoothness of RL were also improved.

## Introduction

Raw lacquer (RL) is an environmentally friendly and renewable material, which is collected from the lacquer tree, and can usually be made into lacquer craft as a lacquer material coated with ceramics, leather, wooden and metal on the surface. In addition, it has been used for thousands of years in history of the East Asia, especially in China^1–3^. It is still used now because of its durability, acid and alkali resistance and high ornamental performance after it cured^4–6^. The compositions of lacquer sap are urushiol (40–80%), water (20–30%), plant gum substance (~ 7%), polysaccharides (~ 5%), laccase (< 1%), and other substance (< 5%)^7–10^.

**Figure 1:**
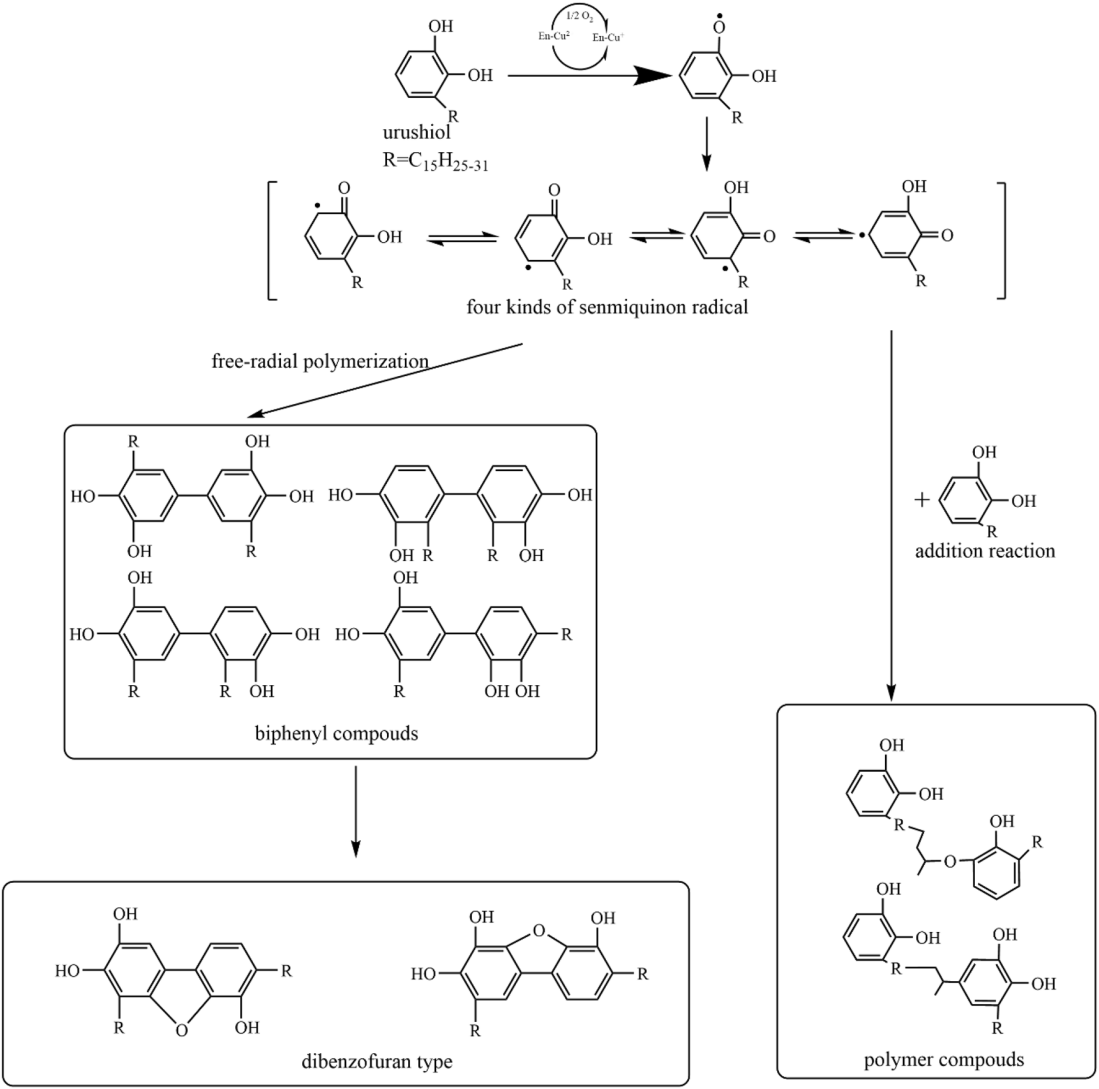
Oxidation mechanism of urushiol catalyzed by laccase.

Urushiol, the main ingredient of lacquer, is a catechol derivative with three different side chain structures^11–14^. Urushiol obtained from *Rhus vernicifera* grown in China has 15 carbons branch chains at 3 positions of the catechol ring^15–17^. Laccase is a phenoloxidase, a protein with four copper ions born in lacquer tree, which catalyzes the oxidation of urushiol through redox interactions between Cu (II) and Cu (I). It is vital that laccase catalyzes in the oxidize urushiol to its phenoxy radical. However, this reaction process needs a relatively hardly condition at the relative humidity about 80–90% and the temperature about 20–30 ℃^14,18^. This is the reason why the lacquer cannot be widely used in the market. The reaction of lacquer is self-oxidization reaction besides catalytic process. At the beginning, laccase as a catalyst is through redox reducing action causing the urushiol oxidized^19,20^. Then while the consistency of the monomer of urushiol is less than 30%, urushiol can react with the unsaturated side chain or senmiquinone radical by itself, as shown in Fig. 1^21^.

Previously, in order to enhance these network structure, some materials were putted into lacquer, such as MWCNTs, nano-silica, organophilic montmorillonite nanocomposites^22–24^. These composites can form new chemical bond with urushiol to improve basic performance of RL. In earlier literatures, researchers found that formaldehyde or amino groups and RL could react to form the network structure, and the urushiol-formaldehyde polymer (UFP) of dry time and other basic performance was improved^25–27^. Inspired by the above research, formaldehyde-urea polymer (FUP) has abundant hydrophilic amino groups and methylol groups, which may have the same effect.

TGA was one of the most common methods for studying thermal degradation analysis. The logarithm of the weight loss rate at different heating rates was plotted against the reciprocal absolute temperature at a given weight loss to obtain the activation energy, and the mechanism was elucidated by the intercept plot^28,29^. Flynn-Wall-Ozawa^30^, the extended Friedman^31^ and the Kissinger–Akahira–Sunose method^32^ are the three commonly used methods. All three methods are derived based on an equation, so these methods have broad applicability. According to this equation, a new equation about thermal degradation kinetics was deduced to research the degradation of lacquer films. Hence, in this study, formaldehyde and urea were used to synthesize FUP, and different amounts of FUP were used to modify the RL, and the basic properties of the RL were tested. TG and SEM were used to analyze the degradation characteristics, the change of activation energy and the surface morphology of RL before and after modification.

## Experiment

### Materials

The lacquer sap was pursued from Chengdu Lacquerware Craft Factory Co., Ltd (Sichuan, China). Formaldehyde solution was 37% aqueous solution, and the concentration of sodium hydroxide (NaOH) solution is 40%. They (including urea) were all analytical grade.

### The preparation of formaldehyde-urea prepolymer (FUP)

The formaldehyde solution and urea placed in a three-necked flask with a thermometer and rotor (the mass ratio of formaldehyde solution to urea was 0.30) and heated in a water bath at 30 ℃. Next adjusted the acid–base with 40% NaOH to make the pH = 8.5. It was heated to 90 °C in less than 40 min, then cooled after 30 min. The FUP sap was obtained.

### The preparation of lacquer film

The lacquer-FUP polymer (LF) was prepared by adding 0%, 1%, 3%, 5% and 7% FUP liquid into the lacquer liquid, and named as RL, LF1, LF2, LF3 and LF4, respectively. Then the LF was put into a beaker and rotated at 40 ℃ for 3 h. Next, the stirred lacquer was coated on tinplate with thickness of 75 μm through the applicator. Finally, the tinplate was put in the humidity chamber (HSP-50B) to dry at 30 ℃ and 80%RH. Then the cured films were put in room environment for a week for subsequent testing.

### Methods

#### The drying time test

The drying time of lacquer was depending on Chinese standard GB/T 1728-2020. The tough-drying meant that when blowing the cotton ball that on the lacquer film gently, if the lacquer didn’t appear cotton silk or can be blown away gently, the time was tough-drying (TD)^33^.

#### Gloss test

The dried lacquer film was tested by the WGG-Y4 glossmeter (Fujian, China) according to Chinese standard GB/T 9754-2007. The principle was to test the reflectance of 60° light on the surface, and the unit of gloss was gloss unit (GU) or gloss (GS).

#### Impact resistance test

The cured lacquer was placed under the CQJ-II instrument (Fujian, China) and tested according to Chinese standard GB/T 1732-2020. The unit of impact resistance was centimeter (cm).

#### Pencil hardness test

Pencil hardness was tested according to Chinese standard GB/T 6739-2006. Gradually increase the hardness of the pencil until there are more than three scratches on the film and record the hardness level of the pencil.

#### Thermal gravimetric analysis

The samples test by Non-isothermal measurements were performed with thermogravimetric (TG) instrument (TG 209F3, Netzsch) at heating rates of 5 ℃/min, 10 ℃/min, 20 ℃/min, 40 ℃/min from 30 to 800 ℃ under nitrogen atmosphere.

#### Scanning electron microscopy (SEM)

The surface morphology of RL and LF were discovered by SEM (Zeiss Sigma 300, Germany).

#### Fourier Transform Infrared Spectrometer (FT-IR) analysis

The FUP, RL and LF2 were tested at room temperature by a spectrometer (Thermo Scientific Nicolet is5, Thermo Fisher). And the prepared samples were tested by KBr compressed into flakes. The wavenumber range is from 400 to 4000 cm^−1^.

#### Kinetic analysis

In the kinetic analysis of solid-state thermal decomposition of polymer materials, the decomposition rate under non isothermal conditions can be expressed as follows:

1 $$\frac{dC}{dt}=kf\left(C\right)$$

where C is the conversion rate ($C=\frac{{W}_{0}-{W}_{t}}{{W}_{0}-{W}_{\infty }}\times 100\%$), W_0_ is the initial weight of the sample, W_t_ is the weight of the sample at time t, W_∞_ is the weight of undecomposable residue.

In Eq. (1), K is the Arrhenius velocity constant, the expression is as follows:

2 $$k=Aexp\left(-\frac{E}{RT}\right)$$

where E is the apparent activation energy, A is the frequency factor, R is the gas constant and T is the absolute temperature.

In Eq. (1), the functional form of f(C) depends on the reaction mechanism. Generally, it can be assumed that f (C) depends only on the reaction degree C, and is independent of temperature T and time t, f (C) can be expressed as follows:

3 $$f(C)={(1-C)}^{n}$$

In Eq. (3), n is the order of reaction. According to Eqs. (1)–(3), it can be concluded that:

4 $$\frac{dC}{dt}=Aexp\left(-\frac{E}{RT}\right){(1-C)}^{n}$$

Considering the constant heating rate $\beta$, Eq. (4) can be expressed as:

5 $$\frac{dC}{dT}=\frac{A}{\beta }exp\left(-\frac{E}{RT}\right){(1-C)}^{n}$$

According to Ozawa's method, Eq. (5) can be deduced as follows^34^:

6 $$Ln\beta =Lg\frac{AE}{RF(C)}-5.331-1.052\frac{E}{R}\times \frac{1}{T}$$

## Results and discussions

### Basic performance analysis

The basic performance of RL and LF was shown in Table 1. With the loading level increased, the TD decreased at first and then increased. Furthermore, the gloss and pencil hardness increased at first and then decreased. The reason of this phenomenon was the presence of some hydrophilicity amino groups in LF, which could change the conditions of lacquer curing. Under low humidity conditions, the water in the lacquer sap evaporated under low vapor pressure, making the lacquer difficult to dry. However, the laccase required a high humidity environment during the redox process.

**Table 1:** The basic performance of RL and LF.

Sample	Loading level	Touch drying (TD)/min	Gloss/GU	Pencil hardness	Impact resistance/cm
RL	–	180	110.3	3H	15
LF1	1%	170	137.6	4H	50
LF2	3%	140	147.3	5H	40
LF3	5%	185	146.6	3H	50
LF4	7%	320	136.5	B	50

Due to the diffusion and penetration of oxygen, the reduced energy of Cu (En-Cu (I)) in the laccase was not oxidized to En-Cu (II), which caused between the Cu (I) and Cu (II) being difficult to converted. However, FUP can improve the work environment of laccase, prompting reactive activity of laccase, accelerating the change between the Cu (I) and Cu (II)^35,36^. In addition, urushiol will complete polymerization in acidic or neutral environment under natural conditions^37^. Therefore, when the additional amount of FUP is small, the amino groups play a crucial role. It can undergo alcoholysis reaction with urushiol groups^33^. With the loading level of FUP increasing, the influence of pH value in the solution was greater than that of the amino group on account of the FUP solution was alkaline. Therefore, the tendency of comprehensive performance of LF appeared a phenomenon of originally becoming better and then becoming worse.

### FT-IR analysis

**Figure 2:**
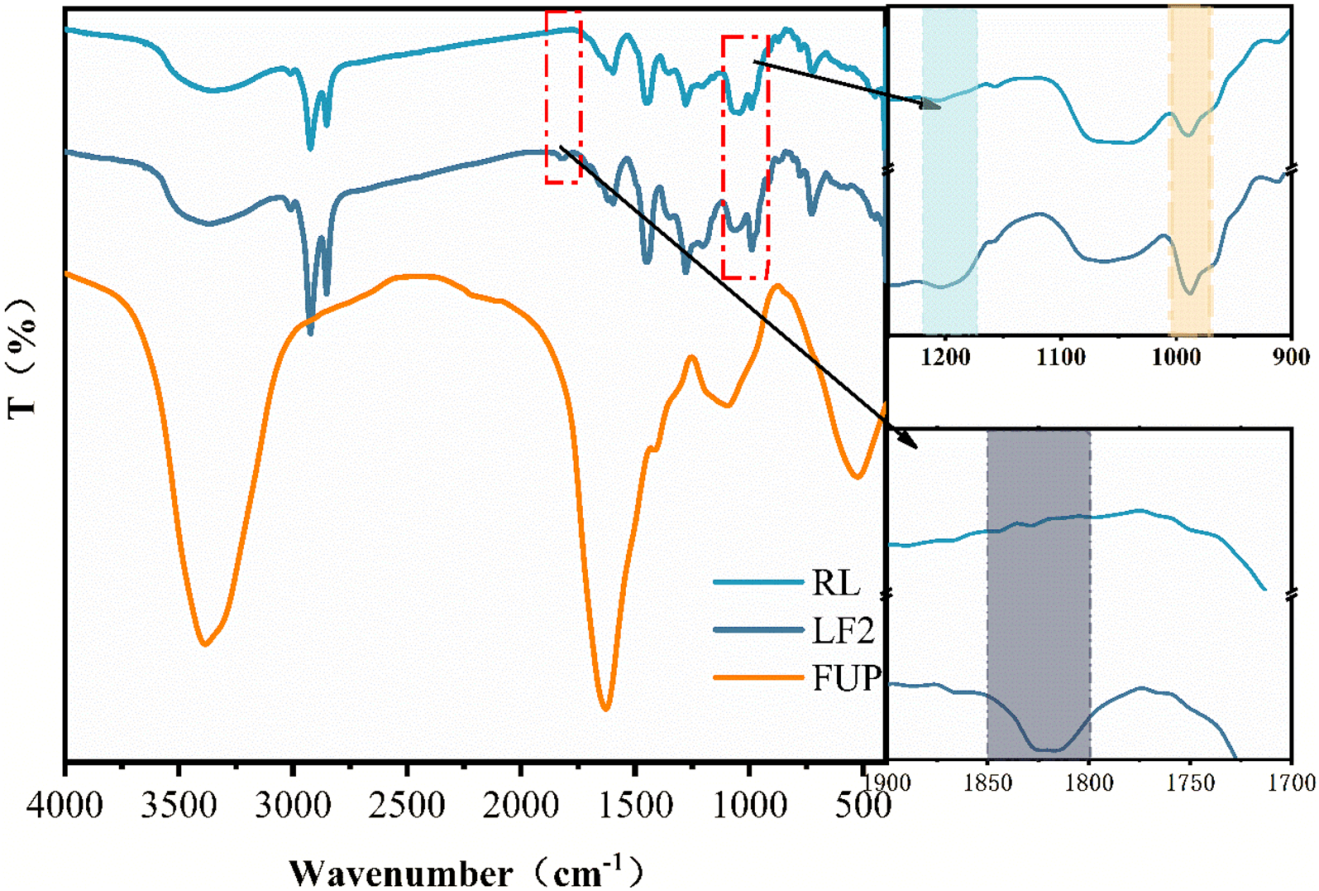
The FT-IR spectra curves of RL, LF2 and FUP.

The FT-IR spectra curves of RL, LF2 and FUP were shown in Fig. 2. In FUP curve, there were mainly three function structures, such as O–H or N–H, C=O and C–N, whose corresponding absorption peaks were at 3200–3700, 1650 and 1000–1200 cm^−1^, respectively. In RL and LF2 curves, there were several absorption peaks as follows. The absorption peaks at 3700–3200, 1360 and 734 cm^−1^ all were O–H tensile vibration. The absorption peaks at 2800–2975, 1475 and 1070–1110 cm^−1^ were caused by the stretching motion of C–H, which were derived from the stretching vibration of C–H on side chain and on catechol ring^38,39^. The peak at 3010 cm^−1^ was the stretching vibration of the C=C bond in the side chain remained.

**Figure 3:**
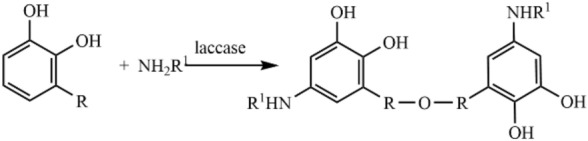
Reaction mechanism of LF.

The peak was assigned to C–O–C vibration at 990 cm^−1^. And according to the peaks were approximately at 1200 cm^−1^ and 1820 cm^−1^, it can be speculated that LF and RL was synthesized C–N bond during the polymerization process and this C–N bond substituted the H on the catechol ring. From the above, it can be inferred that LF can form the single substitution of a C–N bond on the catechol ring, as shown in Fig. 3^40,41^. This C-N bonds in LF2 could enhance network structure when the urushiol was aggregated, thereby enhance the basic performance of LF.

### TG analysis

**Figure 4:**
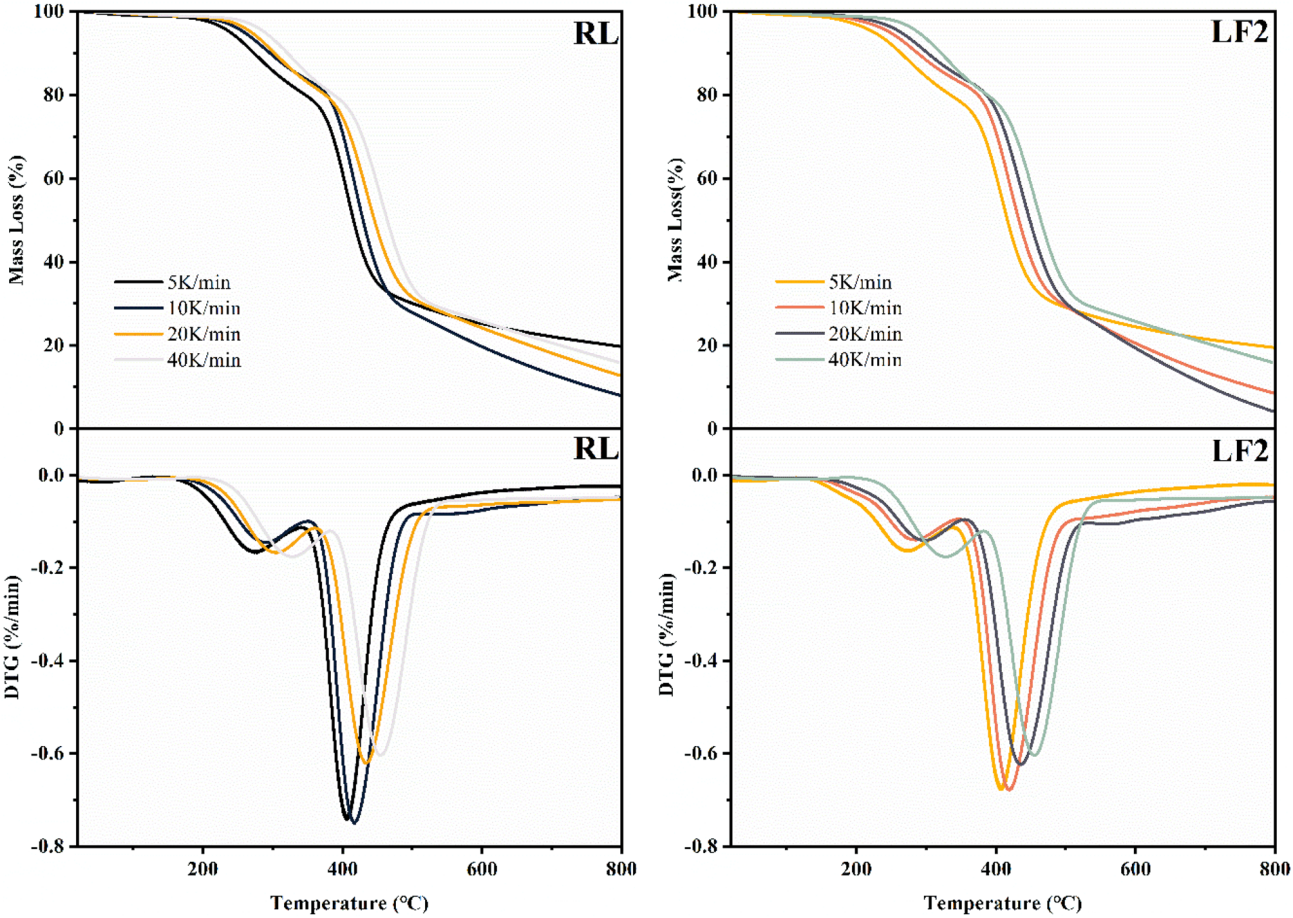
The TG and DTG curves of RL and LF2 four different heating rates.

**Figure 5:**
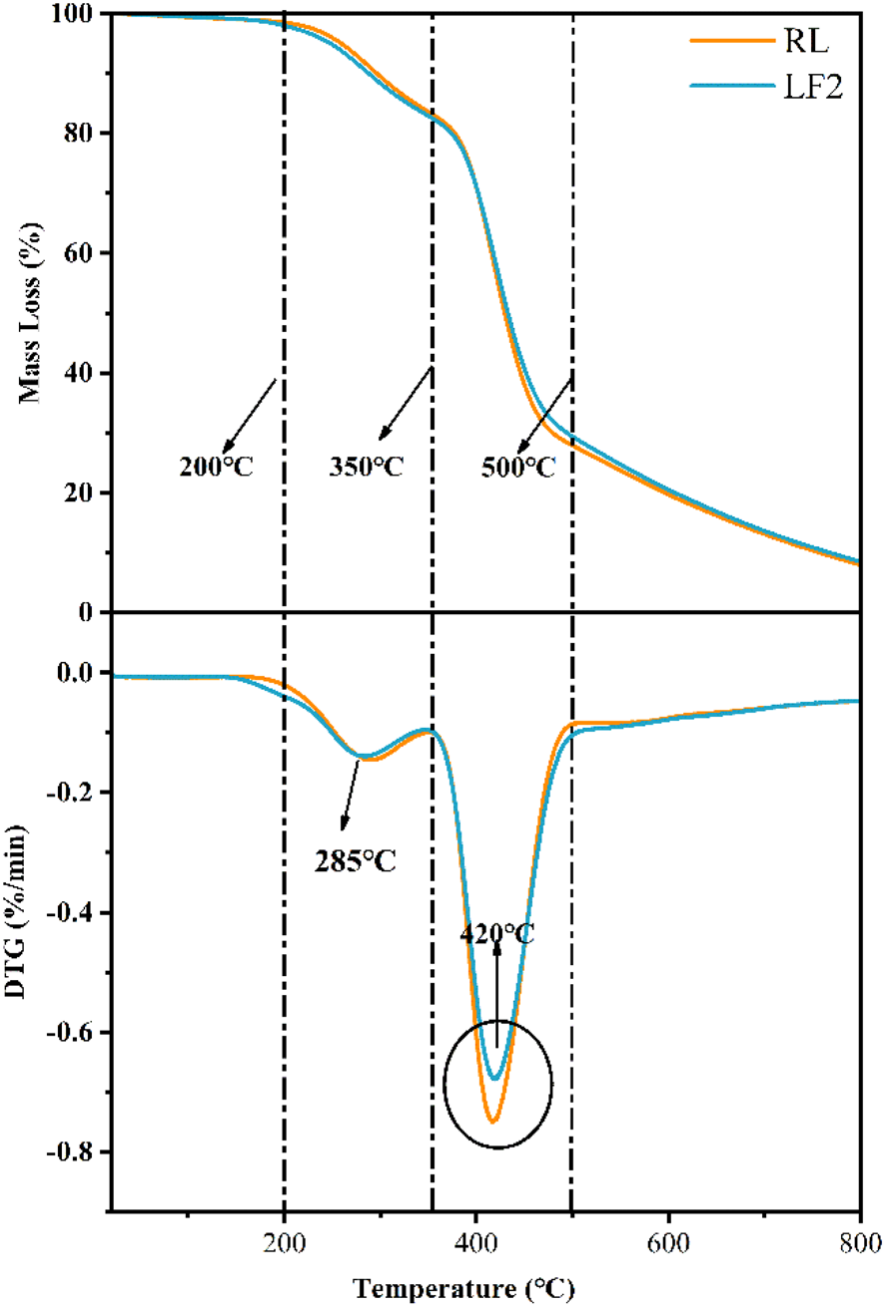
The TG-DTG curves of RL and LF2 with 10 K/min.

The TG curves of RL and LF2 at different heating rates were shown in Fig. 4. And the TG-DTG curves of RL and LF2 with 10 K/min were shown in Fig. 5. Apparently, the degradation of RL and LF had the same tendency. They all had four stages.

The first stage temperature was between 0 and 200 ℃. The mass loss at this stage is mainly the evaporation of water and the moisture content of RL and LF2 were almost the same. The second stage temperature was between 201 and 350 ℃. In this stage, the mass loss is mainly due to the degradation of small molecules. The peak degradation temperature was about 285 ℃^35^. The temperature range for the next stage was from 351 to 500 ℃ and 501–800℃. In this stage, the mass loss was mainly the degradation of glycoprotein, lacquer polysaccharide and lacquer monomer^42,43^.

Thermal parameters of RL and LF2 obtained from TG–DTA curves were shown in the Table 2. It can be found that the mass loss of LF between 201 and  350 ℃ was slightly more than RL. This phenomenon showed that whether it is RL or LF2 in the process of film formation, the urushiol monomer continuously forms dimers and polymers, which led to a decrease in the number of urushiol monomers after curing and film formation. So that in the process of thermal degradation, the difference in film quality loss was very small. It can also be known that most FUP existed in the film in the form of urushiol polymer, and the temperature will not degrade at 350 °C. The degradation of urushiol polymer was mainly in the two stages of 351–500 ℃ and 501–800 ℃, and most of the urushiol polymer in the lacquer film will degrade in these two stages. From the degradation rates of the DTG curves in Table 2 and Fig. 5 at 420 °C, it can be found that the degradation rate of RL was significantly higher than that of LF2, resulting in the degradation of LF2 at this stage was smaller than that of RL. This result led to the fact that the carbon residue of LF2 was greater than RL at 800 °C. The more carbon residues, the stronger the impact resistance of the lacquer film. It can also be confirmed in Table 1.

**Table 2:** Thermal parameters of RL and LF obtained from TG–DTA curves.

Sample	Loading level	Corresponding Mass loss %				Char residual at 800 ℃
		Temperature ℃				
		0–200	201–350	351–500	501–800	
RL	–	1.499	14.929	55.683	19.900	7.989
LF2	3	2.033	15.076	53.505	20.859	8.527

### Calculation of activation energy

**Figure 6:**
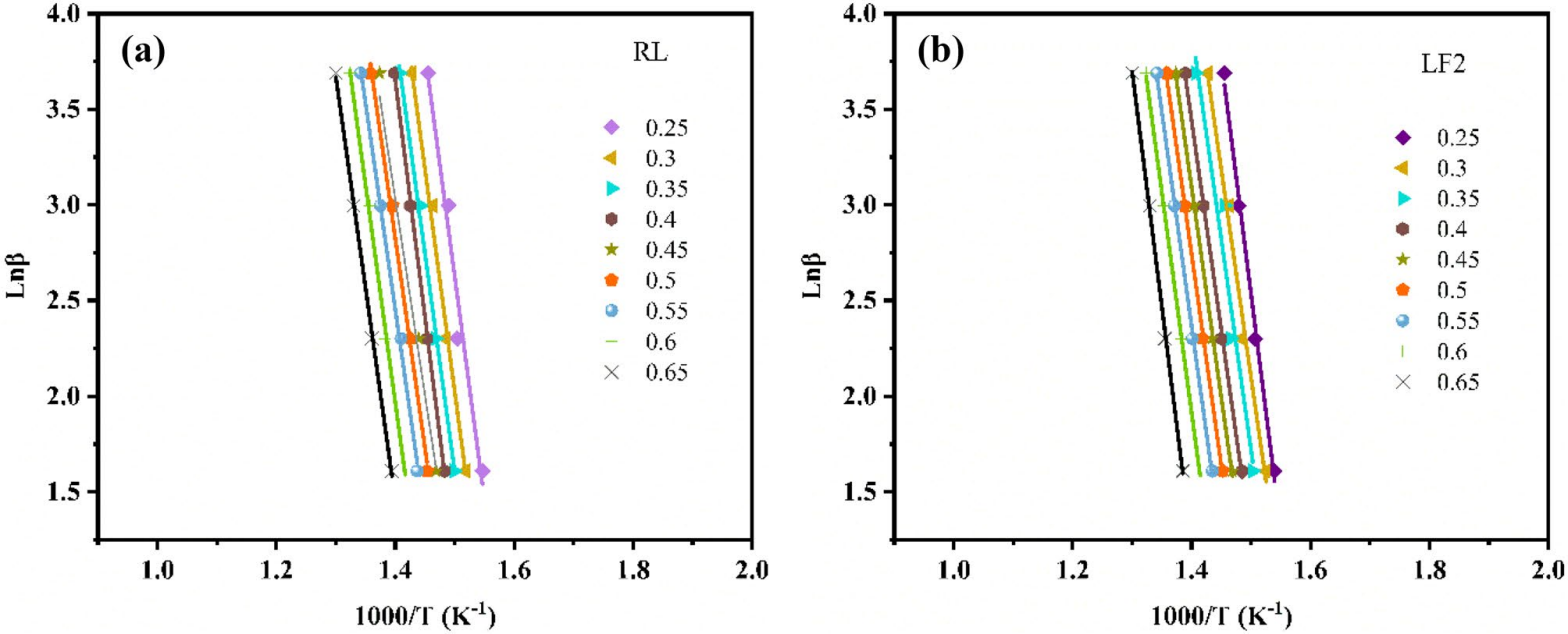
Plots of *ln β* vs 1/T at different conversion rates by Ozawa method (**a**) RL and (**b**) LF2.

Plots of ln β vs 1/T at different conversion rates by Ozawa method were shown in Fig. 6. The activation energy (*E*) obtained by Ozawa method were shown in Table 3^38,44^.

**Table 3:** E values of RL and LF2 calculated by the Ozawa method at different conversion rates.

Conversion rates	RL		LF2	
	E (kJ/mol)	R^2^	E (kJ/mol)	R^2^
0.25	184.713	0.970	164.213	0.961
0.3	183.678	0.992	171.340	0.986
0.35	180.074	0.996	173.703	0.947
0.4	176.667	0.997	173.158	0.998
0.45	161.896	0.977	173.751	0.999
0.5	158.300	0.970	174.723	0.999
0.55	153.439	0.952	176.857	0.999
0.6	144.034	0.905	181.813	0.999
0.65	142.792	0.989	194.798	0.999
Average	165.066		176.040	

**Figure 7:**
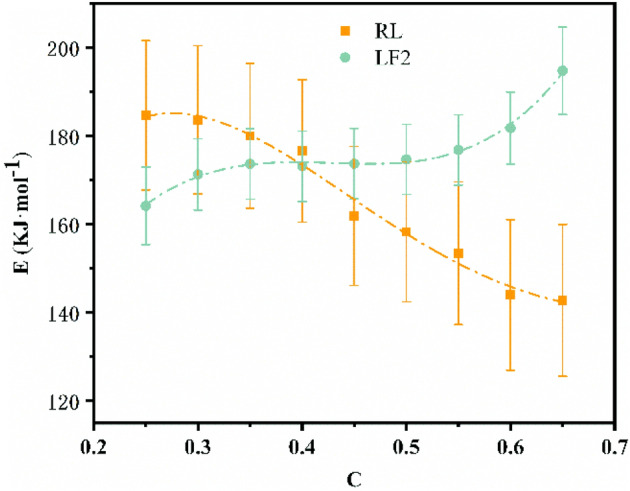
E of RL and LF2 calculated by the Ozawa method at different conversion rates.

The conversion rate in Table 3 intercepted the *E* data between 0.25 and 0.65, because when the conversion rate was before 0.25, it was mainly the degradation of water rather than the degradation of urushiol polymer, so the data cannot be used for reference. After 0.65, the temperature was just at the turning point of 500 °C. After 500 °C, the C–O double bond on the urushiol side chain was mainly thermally degrading. The main products are alkylphenols and alkenylphenols^10^. It was also possible that the C–O double bond in FUP was degrading, resulting in that some of the values cannot be used in the activation energy of thermal degradation of urushiol. When conversion rates are less than 0.25 and greater than 0.65, the correlation coefficient (R^2^) is very low, which cannot be used to calculate *E*. With the increase of conversion rate, the *E* value of RL decreases gradually, while the *E* value of LF2 increases, as shown in Fig. 7. The average* E* value of RL and LF2 was 165.066 and 176.040 kJ/mol, respectively. When the conversion rate is 0.4, the E of LF and RL are basically the same, the corresponding temperature is about 350 °C, which was just the turning point of the thermal degradation rate. The thermally degradable substances were urushiol monomer or urushiol small molecules when the temperature is below 350 ℃. In this range, the *E* of RL was significantly larger than that of LF2, indicating that the small molecules of urushiol in LF2 are more easily degraded at this stage. When the temperature is higher than 350 ℃, the degradation of urushiol polymer was the main factor, and the *E* of LF2 was greater than that of RL in this stage. This was because FUP reacted with urushiol to form aniline analogs and the amino group formed more stable carbon–nitrogen bond with the side chain.

### SEM analysis

**Figure 8:**
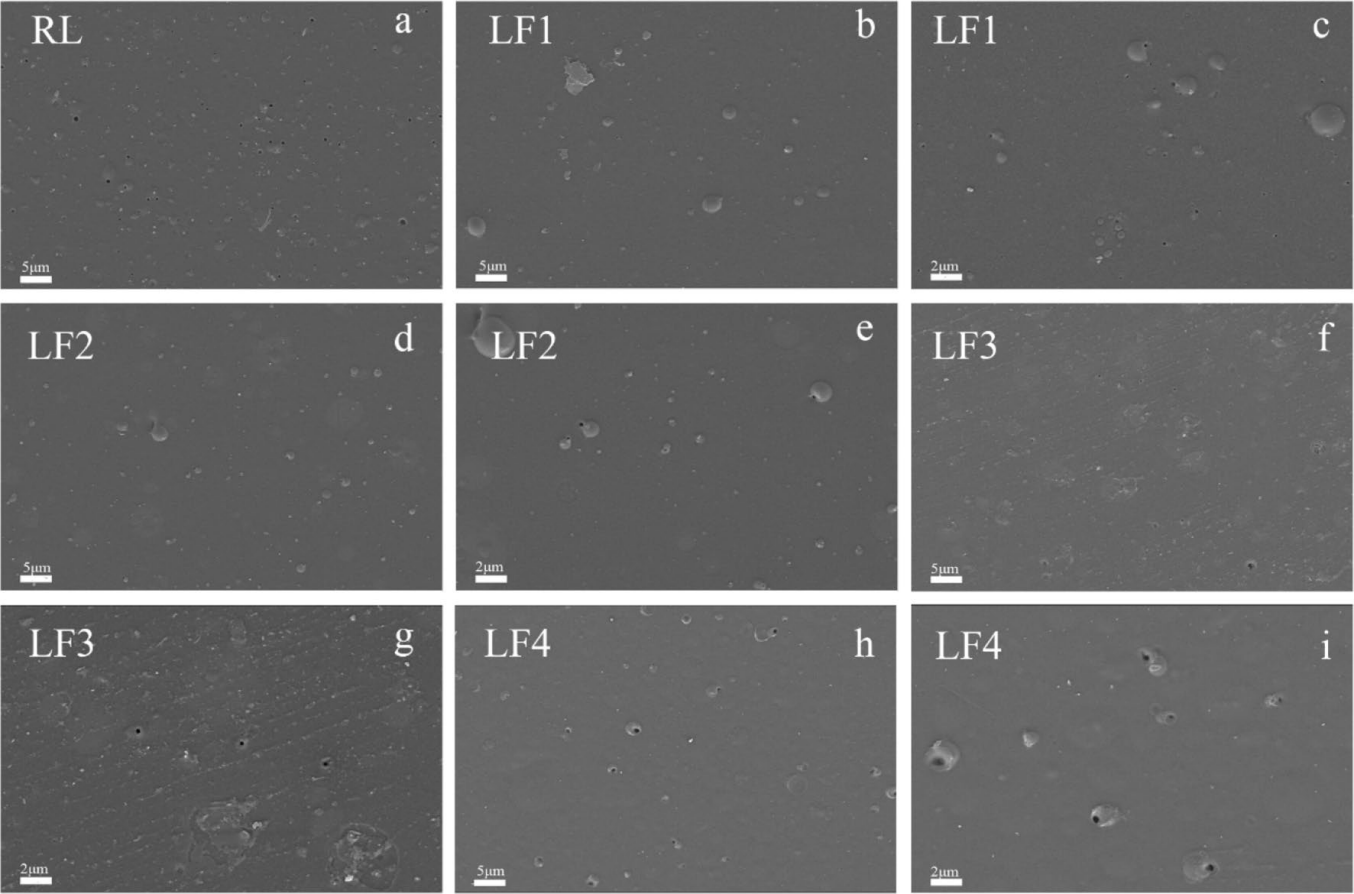
SEM images of membrane of appearance of RL and LF: (**a**) RL; (**b**) and (**c**) LF1; (**d**) and (**e**) LF2; (**f**) and (**g**) LF3; (**h**) and (**i**) LF4.

The SEM images of cured RL and LF were as shown in Fig. 8. It was noticed that there were some caves on the films, which are caused by the evaporation of waterdrops during the drying process^45,46^. In Fig. 8a, the caves on RL is the largest, with a diameter of about 0.5 μm. Compared with RL, LF has smaller caves diameter and smoother surface. As shown in Fig. 8d and e, when the additional amount of FUP was 3%, the surface morphology of LF2 was the smoothest, the diameter of the caves was smaller, and the number was also decreased compared with that of RL. Furthermore, the diameter of caves in Fig. 8h was bigger than RL. This result analysis was basically consistent with the performance of the basic performance in Table 1.

## Conclusion

FUP can significantly improve the performance of RL. When the additional amount of FUP is 3%, the drying time of LF2 is significantly shortened and the gloss, pencil hardness, and impact performance are also significantly enhanced. At the same time, the surface morphology of LF2 was the smoothest, the diameter of the caves was smaller, and the number was also decreased compared with that of RL. This is due to the amino group in FUP can underwent an alcoholic reaction with urushiol. With the increase of conversion rate, the *E* value of RL decreases gradually, while the *E* value of LF2 increases. The average activation energy of LF2 is significantly greater than that of RL. These results demonstrated LF had higher thermal stability and smoother.

## Data Availability

All data generated or analyzed during this study are included in this article (and there are no supplementary materials).
